# Matching Adherence Interventions to Patient Determinants Using the Theoretical Domains Framework

**DOI:** 10.3389/fphar.2016.00429

**Published:** 2016-11-14

**Authors:** Samuel S. Allemann, Robby Nieuwlaat, Bart J. F. van den Bemt, Kurt E. Hersberger, Isabelle Arnet

**Affiliations:** ^1^Pharmaceutical Care Research Group, Department of Pharmaceutical Sciences, University of BaselBasel, Switzerland; ^2^Department of Clinical Epidemiology and Biostatistics, McMaster UniversityHamilton, ON, Canada; ^3^Department of Pharmacy, Radboud University Medical CenterNijmegen, Netherlands; ^4^Department of Pharmacy, Sint MaartenskliniekNijmegen, Netherlands

**Keywords:** medication adherence, intervention, patient determinants, theoretical domains framework, theory

## Abstract

**Introduction:** Despite much research, interventions to improve medication adherence report disappointing and inconsistent results. Tailored approaches that match interventions and patient determinants of non-adherence were seldom used in clinical trials. The presence of a multitude of theoretical frameworks and models to categorize interventions and patient determinants complicated the development of common categories shared by interventions and determinants. We retrieved potential interventions and patient determinants from published literature on medication adherence, matched them like locks and keys, and categorized them according to the Theoretical Domains Framework (TDF).

**Methods:** We identified the most relevant literature reviews on interventions and determinants in a pragmatic literature search, extracted all interventions and determinants, grouped similar concepts to umbrella terms and assigned them to TDF categories. All steps were finalized in consensus discussion between the authors.

**Results:** Sixteen articles (5 with determinants, 11 with interventions) were included for analysis. We extracted 103 interventions and 42 determinants that we divided in 26 modifiable and 16 unmodifiable determinants. All interventions and modifiable determinants were matched within 11 categories (Knowledge; Skills; Social/professional role and identity; Beliefs about capabilities; Beliefs about consequences; Intentions; Memory, Attention and decision processes; Environmental context and resources; Social influences; Emotion; and Behavioral regulation).

**Conclusion:** In published trials on medication adherence, the congruence between interventions and determinants can be assessed with matching interventions to determinants. To be successful, interventions in medication adherence should target current modifiable determinants and be tailored to the unmodifiable determinants. Modifiable and unmodifiable determinants need to be assessed at inclusion of intervention studies to identify the patients most in need of an adherence intervention. Our matched categories may be useful to develop interventions in trials that investigate the effectiveness of adherence interventions.

## Introduction

After 4 decades of research on adherence to medication, the progress is disappointing and adherence remains a fragmented construct. Medication adherence is briefly defined as the behavioral response to an agreed medical recommendation (Sabaté, 2003) and is measured either dichotomously (either one is adherent, or one is not) or continuously. Recently, medication adherence has been defined to consist of three different phases: initiation, implementation, and persistence (Vrijens et al., 2012). Non-adherence is not simply the reverse of adherence. Two patients can be equally non-adherent with respect to measuring adherence, for example take only 60% of their pills. At the same time, the reasons for these patients to be non-adherent may vary widely.

The complexity of the characteristics of adherence was already known by the end of the 1970s (Becker and Maiman, 1975). Despite much research in the 1980s and 1990s, few new insights arose. Research in the 1990s emphasized the influence of patient beliefs about health in general and about illness/medication in particular (Horne and Weinman, 1999). Qualitative research on patients' perspectives started with the new millennium and identified new issues like the quality of the doctor-patient relationship and patient health beliefs (Vermeire et al., 2001). Grossly, five theoretical approaches could be identified that all view non-adherence from a different perspective (Leventhal and Cameron, 1987). The oldest approach is the biomedical model that focuses on dispositional characteristics of the patient such as demographic or personality traits. Operant behavior and social learning theories shifted the focus to the behaviors needed for adherence. In the communication model, the patient seeks expert advice and treatment from the healthcare professional; adherence results from persuasion through effective communication. The rational decision-health belief and reasoned action model generated the patient's perception of risk and motivation for action. Finally, the self-regulative systems theory sees the patients as an active problem solver. Extent and factors of non-adherence have been extensively investigated, and a plethora of strategies to improve adherence was developed, mostly without consistent success (Haynes et al., 2008).

A systematic review of reviews analyzed interventions with regard to theoretical models and found no clear correlation between the effectiveness of interventions that were theory-based and those without an explicit theoretical background (van Dulmen et al., 2007).

The most recent approach, the Theoretical Domains Framework (TDF), was developed to integrate the various behavior change theories. It aimed to simplify the investigation of behavioral problems such as adherence and to facilitate intervention design (Cane et al., 2012).

Success in a complex process like adherence can only be achieved with the integration of many ingredients, and a single obstruction causes failure. This concept is sometimes referred to as the “Anna Karenina principle,” referring to the first sentence in Tolstoy's novel Anna Karenina: “Happy families are all alike. Every unhappy family is unhappy in its own way.” In this regard, it takes one deficient factor to cause non-adherence. Therefore, the purpose of any intervention strategy should be to compensate for each reason causing non-adherence. As acknowledged by others (Hugtenburg et al., 2013; Linn et al., 2013; Müller et al., 2015), it seems thus obvious that a tailored approach is required, i.e., an approach that adapts the keys (interventions) to the locks (patient determinants).

Various attempts to categorize interventions ended up with coarse sections like educational, behavioral, social, and mixed forms (Kripalani et al., 2007) or simple groupings (Haynes et al., 2005). In the field of behavior change research, a recent international consensus developed a framework (the Behavior Change Technique Taxonomy) with 93 behavior change techniques clustered in 16 groups (Michie et al., 2013). Although not specific for medication adherence, the new taxonomy has been used to classify interventions in the field of adherence research (Joost et al., 2014). As behavior change is only one aspect of medication adherence, this unilateral framework appears limited to categorize the sum of all adherence interventions. A broader view on adherence was captured by a Cochrane Review on interventions to improve safe and effective medicines use (Ryan et al., 2014). Interventions were grouped in eight categories: Providing information or education; Facilitating communication and/or decision making; Acquiring skills and competencies; Supporting behavior change; Support; Minimizing risks or harms; Improving quality, and Consumer system participation.

Patient determinants of non-adherence were often categorized according to the five dimensions of non-adherence proposed by WHO (Sabaté, 2003) or variations of these concepts (Baudrant-Boga et al., 2012): Social- and economic-related factors; Health system/health care team-related factors; Therapy-related factors; Condition-related factors, and Patient-related factors.

Matching possible targets for medication adherence to the types of interventions will yield more insight in effective strategies able to overcome the different barriers for medication adherence. To our knowledge, common categories shared by interventions and patient determinants of non-adherence have never been proposed. As a result, interventions for improving adherence and patient determinants were seldom matched in clinical trials. As an example, from 109 studies aimed at improving patient adherence, only 13% reported the assessment of patient determinants at baseline (Haynes et al., 2015). Even though some studies reported tailoring of interventions to patient characteristics, the specific procedure remains often unclear and thus, the results are almost impossible to replicate (Haynes et al., 2015).

In this article, we retrieved potential interventions and patient determinants from published literature on medication adherence and aimed at matching them like locks and keys.

## Goals/aims

- To extract from literature salient (a) interventions intended to improve adherence and (b) related patient determinants of non-adherence.
- To categorize the retrieved (a) interventions and (b) determinants.
- To match (a) and (b).

## Methodology

### Search strategy

Several recent systematic literature reviews exist on interventions and patient determinants of non-adherence. It seemed superfluous to repeat this process and thus, we abstained from a systematic literature search with broad major/MeSH terms such as “patient compliance.” Rather and in order to identify literature with the highest relevance to our aims, we pursued a pragmatic search strategy to identify existing reviews with the terms “intervention” and “determinant” or “factor” which are widely used in conjunction with medication adherence. We combined these specific terms with the established terms “adherence” or “compliance.”

We searched Medline via Pubmed on March 10, 2015 (without time limits) with the following terms and a limit set to reviews:

- intervention^*^[title] AND (improv^*^[title] OR enhanc^*^[title]) AND medication[title] AND (adherence[title] OR compliance[title]).
- (determinant^*^[title] OR factor^*^[title]) AND medication[title] AND (adherence[title] OR compliance[title]).

Titles and abstracts of the search results were screened for relevance by two investigators (SA, IA). To assure a generic view on medication adherence (not restricted to specific diseases, medications or settings), we excluded full-text articles when they investigated:

- single conditions
- single medication groups
- specific providers
- specific target groups
- single intervention
- economic evaluations
- specific adherence measurement methods.

### Data extraction and processing

All extractions were performed in MAXQDA 11 (VERBI GmbH, Berlin, Germany). All steps were performed separately for interventions and determinants.

In the first step, IA and SA reviewed full-texts from the included articles. Both investigators independently extracted items of (a) interventions and (b) determinants for non-adherence and scanned the reference list for additional articles. Investigators were not blinded with regard to authors or journal. The lists were reviewed by both investigators in a consensus discussion and umbrella terms were introduced for items with similar connotations.

In the second step, IA and SA independently matched each intervention to individual determinants. Items that did not match were listed separately.

In the third step, IA and SA assigned the matched interventions and determinants to the 14 domains of the TDF. We chose the TDF because it offers the most recent framework, combines various theoretical models, and has a strong empirical base. We determined consistency among raters performing an interrater reliability analysis using the Kappa statistic.

Disagreements were resolved by discussion until consensus was reached (first and second steps) or by an adjudicator (third step).

## Results

### Literature search

**Figure 1:**
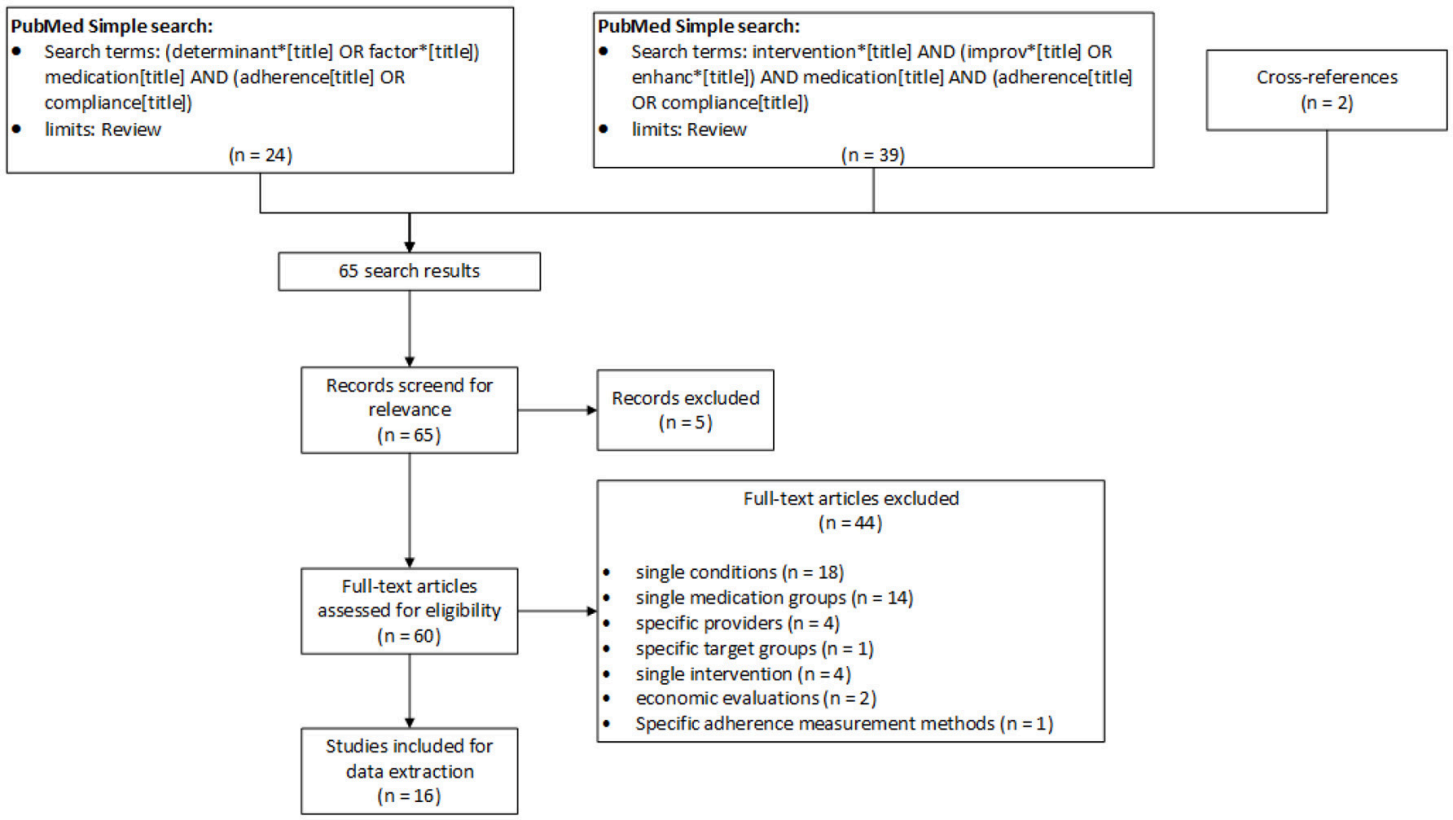
**PRISMA flow diagram (Moher et al., 2009) for study selection process**.

A total of 65 articles were obtained (Figure 1). Two articles were updated versions of previous Cochrane analyses (Haynes et al., 2008; Schedlbauer et al., 2010). Screening of the reference lists yielded two additional articles that were included in the review (Vermeire et al., 2001; Ryan et al., 2014). Five articles were excluded after screening of titles and abstracts because they were not relevant to our aims. After full-text screening, 44 articles were excluded because they investigated (a) single conditions (18; schizophrenia, psychiatric disorder, transplantation, diabetes, hypertension, Parkinson, inflammatory bowel, rosacea); (b) single medication groups (14; antidepressant, cardiovascular, heart failure, antipsychotic, osteoporosis, hypoglycemic and lipid lowering agents); (c) specific providers (4; pharmacist, physician, nurse); (d) specific target groups (1; children); (e) single intervention (4; HIT, technology-mediated, cultural responsive, electronic reminders); (f) economic evaluations (2); (g) adherence measurement methods (1; electronically compiled drug dosing history).

The final set included 16 articles (11 with interventions, 5 with determinants, Table 1).

**Table 1:** **Overview of included literature**.

**Title [original language]**	**Authors**	**Year**	**Type of study**	**Country**	**Conditions**	**Medications**
**DETERMINANTS**						
Thinking differently the patient medication compliance: From an injunctive posture to a working alliance between the patient and the healthcare provider—Concepts and determinants [French] (Baudrant-Boga et al., 2012)	Baudrant-Boga M, Lehmann A, Alleneta A	2012	General Review	France	ns	ns
The impact of medication regimen factors on adherence to chronic treatment: a review of literature (Ingersoll and Cohen, 2008)	Ingersoll KS, Cohen J	2008	Review	USA	Chronic illness (asthma, diabetes, HIV disease, and hypertension/cardiovascular disease, mental disorders, pain, and other diseases), contraception	ns
Medication non adherence—predictive factors and diagnostic [German] (Schäfer-Keller et al., 2010)	Schäfer-Keller P, Garzoni D, Dickenmann M, De Geest S	2010	General Review	Switzerland	ns	ns
Medication Adherence: Factors influencing compliance with prescribed medication plans (Vlasnik et al., 2005)	Vlasnik JJ, Aliotta SL, DeLor B	2005	General Review	USA	ns	ns
A systematic literature review of psychosocial and behavioral factors associated with initial medication adherence: a report of the ISPOR medication adherence and persistence special interest group (Zeber et al., 2013)	Zeber JE, Manias E, Williams AF, Hutchins D, Udezi WA, Roberts CS, Peterson AM	2013	Systematic Review	USA	Acute and chronic conditions (hypertension, diabetes, cardiovascular disease, depression or anxiety, Asthma, osteoporosis, epilepsy, cancer, multiple sclerosis, other diseases)	ns
**INTERVENTIONS**						
A review of interventions used to improve adherence to medication in older people (Banning, 2009)	Banning M	2009	Review	United Kingdom	ns	ns
Interventions for enhancing medication adherence (Haynes et al., 2005)	Haynes RB, Yao X, Degani A, Kripalani S, Garg A, McDonald HP	2005	Systematic Review	Canada	Medical disorders (including psychiatric), but not addiction	Self-administered
Interventions for enhancing medication adherence (Haynes et al., 2008)	Haynes RB, Ackloo E, Sahota N, McDonald HP, Yao X	2008	Systematic Review	Canada	Medical disorders (including psychiatric), but not addiction	Self-administered
Interventions to enhance medication adherence in chronic medical conditions (Kripalani et al., 2007)	Kripalani S, Yao X, Haynes RB	2007	Systematic Review	Canada	Chronic medical conditions	Self-administered
Interventions to enhance patient adherence to medication prescriptions interventions to enhance patient adherence to medication prescriptions (McDonald et al., 2002)	McDonald HP, Garg AX, Haynes RB	2002	Scientific Review	Canada	Medical or psychiatric disorders (hypertension, schizophrenia or acute psychosis, asthma, chronic obstructive pulmonary disease, depression, HIV, diabetes, rheumatoid arthritis, epilepsy, hyperlipidemia and cardiovascular disease, acute infections)	Self-administered
Interventions for enhancing medication adherence (Nieuwlaat et al., 2014)	Nieuwlaat R, Wilczynski N, Navarro T, Hobson N, Jeffery R, Keepanasseril A, Agoritsas T, Mistry N, Iorio A, Jack S, Sivaramalingam B, Iserman E, Mustafa RA, Jedraszewski D, Cotoi C, Haynes RB	2014	Systematic Review	Canada	Medical disorders (including psychiatric), but not addiction	Self-administered
Interventions to improve safe and effective medicines use by consumers: an overview of systematic reviews (Ryan et al., 2014)	Ryan R, Santesso N, Lowe D, Hill S, Grimshaw J, Prictor M, Kaufman C, Cowie G, Taylor M	2014	Overview of reviews	Australia	Acute and chronic diseases	ns
Medication non-adherence among older adults: a review of strategies and interventions for improvement (Schlenk et al., 2004)	Schlenk EA, Dunbar-Jacob J, Engberg S.	2004	General Review	USA	ns	ns
Interventions to improve medication compliance in older patients living in the community (van Eijken et al., 2003)	van Eijken M, Tsang S, Wensing M, de Smet PA, Grol RP.	2003	Systematic Review	The Netherlands	ns	ns
Patient adherence to treatment: three decades of research. A comprehensive review (Vermeire et al., 2001)	Vermeire E, Hearnshaw H, Van Royen P, Denekens J.	2001	Comprehensive Review	Belgium	ns	ns
Interventions to improve medication adherence in people with multiple chronic conditions: a systematic review (Williams et al., 2008)	Williams A, Manias E, Walker R.	2008	Systematic Review	Australia	Three or more chronic conditions	ns

*ns: not specified*.

We extracted 103 different interventions, including variations of the same concept such as different forms of reminders. We extracted 42 determinants that we divided into 26 modifiable and 16 unmodifiable determinants. We defined modifiable determinants as factors that may be changed by interventions (such as knowledge or behaviors) and unmodifiable determinants as those that are unchangeable (such as age). Some unmodifiable determinants may appear modifiable at first sight, such as level of education or employment situation. However, those determinants are not targeted by the adherence interventions, albeit they may influence the choice of an appropriate intervention. Thus, unmodifiable determinants (Box 1) were not included in the matching procedure.

Box 1 **Unmodifiable determinants of non-adherence**

• *Age* (Vlasnik et al., 2005; Ingersoll and Cohen, 2008; Schäfer-Keller et al., 2010; Baudrant-Boga et al., 2012)

• *Gender* (Vlasnik et al., 2005)

• *Level of education* (Vlasnik et al., 2005; Ingersoll and Cohen, 2008) (literacy)

• *Employment situation* (Vlasnik et al., 2005)

• *Financial situation* (Vlasnik et al., 2005; Baudrant-Boga et al., 2012) (socioeconomic status, lack of insurance, income, material resources)

• *Insurance type/coverage* (Zeber et al., 2013)

• *Ethnicity and culture* (Vlasnik et al., 2005; Schäfer-Keller et al., 2010; Baudrant-Boga et al., 2012; Zeber et al., 2013) (language difficulties, race, immigration status)

• *Housing situation/living situation* (Vlasnik et al., 2005; Baudrant-Boga et al., 2012; Zeber et al., 2013) (lack of fixed address, living alone, marital status)

• *Cognitive impairment* (Vlasnik et al., 2005; Ingersoll and Cohen, 2008; Baudrant-Boga et al., 2012)

• *Illness chronicity* (Zeber et al., 2013)

• *Illness severity* (Vlasnik et al., 2005; Schäfer-Keller et al., 2010; Baudrant-Boga et al., 2012; Zeber et al., 2013) (absence, reduction, disappearance or fluctuation of symptoms)

• *Polymorbidity* (Zeber et al., 2013)

• *Change of therapy* (Vlasnik et al., 2005; Schäfer-Keller et al., 2010)

• *History of non-adherence* (Vlasnik et al., 2005)

• *Past treatment response* (Zeber et al., 2013)

• *Duration of treatment* (Schäfer-Keller et al., 2010; Baudrant-Boga et al., 2012).

### Matching procedure

From the original 14 domains of the TDF, 11 suffice to categorize our 103 interventions and 26 modifiable determinants (Table 2). No intervention or determinant could be assigned to the three original domains “Optimism,” “Reinforcement,” and “Goals.” Because heterogeneous interventions and determinants were included in the domain “Environmental context and resources,” we created the subdomains “Regimen,” “Adverse events,” “Integration and coordination of care,” and “Financial aspects” (Table 3).

**Table 2:** **Final 11 categories of the TDF with corresponding definitions sufficient to categorize interventions and patient determinants**.

**Category**	**Interventions and determinants focusing on …**
1. Knowledge	…the awareness of the existence of something
2. Skills	…the ability or proficiency acquired through practice
3. Social/professional role and identity	…behaviors and displayed personal qualities of an individual in a social or work setting
4. Beliefs about capabilities	…the acceptance of the truth, reality, or validity about an ability, talent, or facility that a person can put to constructive use
5. Beliefs about consequences	…The acceptance of the truth, reality, or validity about outcomes of a behavior in a given situation
6. Intentions	…the conscious decision to perform a behavior or a resolve to act in a certain way
7. Memory, attention and decision processes	…the ability to retain information, focus selectively on aspects of the environment and choose between two or more alternatives
8. Environmental context and resources	…any circumstance of a person's situation or environment that discourages or encourages the development of skills and abilities, independence, social competence, and adaptive behavior
9. Social influences	…those interpersonal processes that can cause individuals to change their thoughts, feelings, or behaviors
10. Emotion	…the complex reaction pattern, involving experiential, behavioral, and physiological elements, by which the individual attempts to deal with a personally significant matter or event
11. Behavioral regulation	…anything aimed at managing or changing objectively observed or measured actions

**Table 3:** **Matched adherence interventions and patient determinants according to 11 TDF categories**.

**Interventions**	**Determinants (examples)**
**KNOWLEDGE**	
• Educate patients (Kripalani et al., 2007; Ryan et al., 2014)	
° Provide information, e.g.,	
• Provide copy of medical record (van Eijken et al., 2003)	
• Provide medication charts/fact sheets (Schlenk et al., 2004; Haynes et al., 2005, 2008; Ryan et al., 2014)	
° Provide instruction, e.g.,	
• Visual, verbal, written materials (van Eijken et al., 2003; Haynes et al., 2005, 2008; Ryan et al., 2014)	
• Self-help workbook (McDonald et al., 2002)	
• Programmed learning (McDonald et al., 2002; Haynes et al., 2005, 2008)	
• Adequate labeling (Vermeire et al., 2001) • Icon-labeled medication containers (Banning, 2009) • Harm-reduction training (Ryan et al., 2014) • Counsel, give advice about treatment ° Benefits (Haynes et al., 2005, 2008; Kripalani et al., 2007) ° Importance (Haynes et al., 2005, 2008) ° Goal (Haynes et al., 2005, 2008) ° Mode of action (Haynes et al., 2005, 2008; Kripalani et al., 2007) ° Causes of low effect (Haynes et al., 2005, 2008) ° Correct use (of medication/device; Vermeire et al., 2001; Haynes et al., 2005, 2008; Kripalani et al., 2007; Ryan et al., 2014) ° Medication adherence (Haynes et al., 2005, 2008; Kripalani et al., 2007) • Discuss knowledge about treatment (Ryan et al., 2014)	•*knowledge about therapy and devices* (Vlasnik et al., 2005; Baudrant-Boga et al., 2012; know-how)
• Counsel, give advice about ° Target disease (Haynes et al., 2008) ° Symptoms (McDonald et al., 2002; Haynes et al., 2005, 2008; Kripalani et al., 2007) ° Health (Haynes et al., 2005, 2008) •Discuss knowledge about health (Ryan et al., 2014)	•*Knowledge about illness* (Vlasnik et al., 2005; Baudrant-Boga et al., 2012; insight into the disease, understanding of the need for treatment)
**SKILLS**	
• Swallowing training (Kripalani et al., 2007) • Easy-to-use packaging (Vermeire et al., 2001) • Physiotherapy (Haynes et al., 2005) • Self-administration training (Ryan et al., 2014)	•*Physical difficulties* (Vlasnik et al., 2005; Baudrant-Boga et al., 2012; swallowing difficulties, difficulties in handling small tablets or opening drug containers, visual impairment)
•Self-management skills (Kripalani et al., 2007; Ryan et al., 2014) • Problem-solving training (McDonald et al., 2002) • Inpatient self-medication programs (Schlenk et al., 2004)	•*Health literacy*
• Communication skills training (Vermeire et al., 2001; Ryan et al., 2014)	• *Communication skills* (Vlasnik et al., 2005; Schäfer-Keller et al., 2010; Baudrant-Boga et al., 2012)
**SOCIAL/PROFESSIONAL ROLE AND IDENTITY**	
• Contract (Kripalani et al., 2007) • Improve relationship (Vermeire et al., 2001; Kripalani et al., 2007; Ryan et al., 2014) ° consumer involvement • Encouraging doctor-patient co-operation • Patient-centeredness • Taking into account of spiritual and psychological dimensions which may be of primary importance to patients • Accurate recognition of the patient’s problem by the health care provider	• *Relationship patient—health care professional* (Vlasnik et al., 2005; Schäfer-Keller et al., 2010; Baudrant-Boga et al., 2012; Zeber et al., 2013; therapeutic alliance)
**BELIEFS ABOUT CAPABILITIES**	
• Patient empowerment (Haynes et al., 2005, 2008; Kripalani et al., 2007)	• *Beliefs about self* (Vlasnik et al., 2005; Baudrant-Boga et al., 2012; perceived importance of self-care)
**BELIEFS ABOUT CONSEQUENCES**	
• Cognitive restructuring (Haynes et al., 2005, 2008) • Cognitive behavioral therapy (Kripalani et al., 2007; Haynes et al., 2008; Ryan et al., 2014)	
• Discuss ° Beliefs (Ryan et al., 2014) ° Barriers (Ryan et al., 2014) ° Ambivalence to treatment (Haynes et al., 2005, 2008) ° Adherence (Haynes et al., 2008)	• *Beliefs about treatment* (Vlasnik et al., 2005; Baudrant-Boga et al., 2012; Zeber et al., 2013; faith in medication, concerns about taking drugs, fear of addiction, preferences, perceived harms vs. benefits)
• Discuss ° Beliefs (Ryan et al., 2014) ° Barriers (Ryan et al., 2014) ° Stigma (Haynes et al., 2005, 2008)	• *Beliefs about health* (Vlasnik et al., 2005; Baudrant-Boga et al., 2012; Zeber et al., 2013; anger, denial of the illness or its significance, apathy, confidence)
	• *Beliefs about health care system* (Vlasnik et al., 2005; Zeber et al., 2013; trust in health care system)
**INTENTIONS**	
• Counseling about lifestyle (Kripalani et al., 2007) ° Diet (Schlenk et al., 2004; Haynes et al., 2005, 2008) ° Exercise (Haynes et al., 2005, 2008) ° Smoking (Haynes et al., 2005, 2008)	• *Lifestyle* (Vlasnik et al., 2005; Baudrant-Boga et al., 2012; Zeber et al., 2013; stress, substance abuse, smoking, alcohol use)
• Rewards (McDonald et al., 2002; Haynes et al., 2005, 2008; Kripalani et al., 2007; Nieuwlaat et al., 2014; Ryan et al., 2014) ° Material ° Monetary • Motivational interviewing (Ryan et al., 2014) • Action plans (Ryan et al., 2014)	• *Motivation* (Vlasnik et al., 2005; Ingersoll and Cohen, 2008; Schäfer-Keller et al., 2010; Baudrant-Boga et al., 2012; readiness to change)
**MEMORY, ATTENTION, AND DECISION PROCESSES**	
• Reminders ° Postcard (Ryan et al., 2014) ° Mailings (McDonald et al., 2002) ° Prescription refill (McDonald et al., 2002; Haynes et al., 2005, 2008) ° Telephone-linked computer system McDonald et al., 2002; van Eijken et al., 2003; Haynes et al., 2005, 2008; Kripalani et al., 2007; Nieuwlaat et al., 2014; Ryan et al., 2014) ° Appointment (McDonald et al., 2002; Haynes et al., 2005, 2008; Ryan et al., 2014) ° Phone call (McDonald et al., 2002; van Eijken et al., 2003; Nieuwlaat et al., 2014; Ryan et al., 2014) ° Mobile text messages (Nieuwlaat et al., 2014) ° Alarms (Ryan et al., 2014) • Organizers (Vermeire et al., 2001; Ryan et al., 2014) • Unit-of-use dispensing (Haynes et al., 2005, 2008) • Automated dispenser (van Eijken et al., 2003) • Directly observed therapy (Haynes et al., 2005, 2008; Kripalani et al., 2007; Ryan et al., 2014) • Patient diary (Kripalani et al., 2007; Nieuwlaat et al., 2014) • Reminder pill packaging (Haynes et al., 2005, 2008; Kripalani et al., 2007; Ryan et al., 2014) • Disposing of excessive medication (Haynes et al., 2005, 2008; Kripalani et al., 2007) • Feedback on medication use (Vermeire et al., 2001; Haynes et al., 2005, 2008; Kripalani et al., 2007)	• *Forgetfulness* (Vlasnik et al., 2005; Ingersoll and Cohen, 2008; Schäfer-Keller et al., 2010)
**ENVIRONMENTAL CONTEXT AND RESOURCES**	
**Regimen**	
• Tailor treatment to daily habits (Vermeire et al., 2001; Schlenk et al., 2004; Haynes et al., 2005, 2008) • Simplified dosing regimens (Vermeire et al., 2001; Haynes et al., 2005; Kripalani et al., 2007; Ryan et al., 2014)	• *Intrusiveness* (Ingersoll and Cohen, 2008; inconvenience, attention to routine)
• Reducing the frequency of dosing (Vermeire et al., 2001; Haynes et al., 2005, 2008; Kripalani et al., 2007; Ryan et al., 2014) • Combination pills (Nieuwlaat et al., 2014)	• *Pill burden* (Vlasnik et al., 2005; Ingersoll and Cohen, 2008; Baudrant-Boga et al., 2012; units per dose, doses per day)
• Changing the medication formulation (Haynes et al., 2005, 2008; Ryan et al., 2014)	• *Specificity of regimen* (Vlasnik et al., 2005; Ingersoll and Cohen, 2008; Baudrant-Boga et al., 2012; Zeber et al., 2013; time-dependence, storage and food restrictions, formulation, time needed for treatment)
**Adverse events**	
• Counseling ° Safety (Haynes et al., 2005, 2008) ° Adverse events (Kripalani et al., 2007; Haynes et al., 2008) • Delayed antibiotic prescriptions (Ryan et al., 2014)	• *Adverse events* (Vlasnik et al., 2005; Ingersoll and Cohen, 2008; Schäfer-Keller et al., 2010; Zeber et al., 2013; treatment-associated adverse reactions)
**Integration and coordination of care**	
• Collaborative care (Vermeire et al., 2001; Banning, 2009) • Reduced frequency of visits (Haynes et al., 2008) • Liaising with general practitioner (Haynes et al., 2005, 2008) • Pharmaceutical care services (Haynes et al., 2005, 2008; Ryan et al., 2014) ° Medicine reconciliation (Banning, 2009; Ryan et al., 2014) (recognition of medication discrepancies) ° Medicines review (Ryan et al., 2014) ° Review illness history (Haynes et al., 2005, 2008) ° Care plan (Ryan et al., 2014) ° Multisystemic therapy (Haynes et al., 2008) • clarify responsibility for administration of therapy (Vlasnik et al., 2005)	• *Number of providers* (Vlasnik et al., 2005; Ingersoll and Cohen, 2008; Zeber et al., 2013)
• Increase the convenience of care (Haynes et al., 2005, 2008) ° Short waiting time (Vermeire et al., 2001) ° Short intervals between appointment (Vermeire et al., 2001) • Provision of therapy at worksite (Haynes et al., 2005, 2008) • Home visits (Kripalani et al., 2007; Ryan et al., 2014)	• *Access to care* (Vlasnik et al., 2005; Baudrant-Boga et al., 2012; Zeber et al., 2013; difficulties in getting prescriptions filled, lack of transportation)
• Discharge planning (Banning, 2009) • (Post-discharge) Follow-up (Schlenk et al., 2004; Haynes et al., 2005, 2008; Ryan et al., 2014) • Periodic reinforcement (Williams et al., 2008) • Mass mailings (Ryan et al., 2014)	• *Continuity of care* (Baudrant-Boga et al., 2012)
• Remote internet-based treatment support (Nieuwlaat et al., 2014)	• *Availability of health care professionals* (Schäfer-Keller et al., 2010; Baudrant-Boga et al., 2012; overworked personnel, organization of care, quality of care network)
	• *Prescribing errors* (Zeber et al., 2013)
**Financial aspects**	
• Financial incentives (Haynes et al., 2005, 2008; Ryan et al., 2014) • Co-payments (Vermeire et al., 2001; McDonald et al., 2002; Ryan et al., 2014)	• *Cost of treatment* (Vlasnik et al., 2005; Schäfer-Keller et al., 2010; Zeber et al., 2013; inability to afford medication, cost of care, out-of-pocket medication expenses)
**Social influences**	
• (Culturally modified) family intervention (McDonald et al., 2002; Haynes et al., 2005, 2008; Kripalani et al., 2007; Ryan et al., 2014) • Social support ° Lay health mentoring (Haynes et al., 2005, 2008) ° (Couple-focused) group programs (Haynes et al., 2005, 2008; Kripalani et al., 2007; Ryan et al., 2014)	• *Social/family support* (Vlasnik et al., 2005; Baudrant-Boga et al., 2012; Zeber et al., 2013; disrupted family structure)
**EMOTION**	
• Psychological therapy (Haynes et al., 2005, 2008; Williams et al., 2008; Banning, 2009; Ryan et al., 2014; Nieuwlaat et al., 2014) • Crisis intervention (Haynes et al., 2005, 2008)	• *Psychological problems* (Vlasnik et al., 2005; Ingersoll and Cohen, 2008; Schäfer-Keller et al., 2010; Baudrant-Boga et al., 2012; depression, apathy, psychosis)
**BEHAVIORAL REGULATION**	
• Point-of-care testing (Kripalani et al., 2007) • Self-monitoring ° Treatment (Haynes et al., 2005, 2008; Ryan et al., 2014) ° Symptoms (McDonald et al., 2002)	• *Monitoring of treatment* (Vlasnik et al., 2005)

*Items were extracted from literature (103 interventions and 25 determinants). Examples and synonyms from the literature are given in brackets*.

The interrater reliability was substantial with a Cohen's Kappa of 0.7 (95% CI 0.5–0.9, *p* <0.001).

## Discussion

Based on a literature review, we were able to match 103 adherence interventions and 26 patient determinants within 11 common categories. The fact that interventions were more diverse than patient determinants is not surprising, as there is usually more than one way to target a single determinant. In a previous review on patient determinants, the authors grouped similar contents together and ended up with 40 heterogeneous umbrella terms (Kardas et al., 2013). What appears nearly identical to our 42 patient determinants is slightly different, since no overlap existed with our determinants for 6 of the 40 umbrella terms (“Social stigma of disease”; “Prescription coverage”; “Prescription by a specialist”; “Certain diagnoses/indications”; “Drug type,” and “Well organized treatment”). A subset of patient determinants must be considered unmodifiable, such as age, gender, or culture. In our view, this distinction is essential for the choice of adherence interventions. In order to be effective, we postulate that interventions have on one hand to *target* current *modifiable* patient determinants and on the other hand to be *tailored* to the *unmodifiable* patient determinants. This lack of distinction among the patient determinants in previous literature may explain partly why no meta-analysis could demonstrate an overall benefit of interventions aimed at enhancing adherence (Nieuwlaat et al., 2014). Further research is needed to investigate if adherence is improved when the intervention is matched to the patient determinants for non-adherence according to our matching list.

The importance of tailoring interventions to patient characteristics has been acknowledged previously (Hugtenburg et al., 2013; Linn et al., 2013; Müller et al., 2015). To our knowledge, no published framework aimed to match interventions and patient determinants of non-adherence. Specific toolboxes for tailored interventions cover a restricted number of interventions or patient determinants (Staring et al., 2006; Herborg et al., 2008). Their intended use is the application in daily practice where a workable toolbox trumps a comprehensive framework. The 5 WHO dimensions that could be used to classify both interventions and patient determinants are too coarse to provide meaningful guidance (Sabaté, 2003). The TDF has been the most recent and complete effort to develop theory-informed behavior change interventions (French et al., 2012) or assess the underlying theoretical constructs of interventions (Little et al., 2015). Because it was not specifically developed for interventions and patient determinants of non-adherence, some adaption was warranted. While we were able to assign all interventions and patient determinants to one of the domains, we did not use 3 of the original 14 domains: “Optimism,” “Reinforcement,” and “Goals”. “Optimism” (i.e., the confidence that things will happen for the best or that desired goals will be attained) may theoretically differ from beliefs, but we chose not to differentiate between the two concepts for practical reasons. By definition, “Reinforcement” (i.e., increasing the probability of a response by arranging a dependent relationship, or contingency, between the response and a given stimulus) can only apply to interventions. Hence, we were not able to use this domain for shared interventions and determinants. Instead, we assigned interventions belonging to the “Reinforcement” domain to “Intentions”. “Goals” (i.e., mental representations of outcomes or end states that an individual wants to achieve) also overlaps with “Intentions” and we chose not to differ between the two concepts.

Some of the extracted interventions did not target patient determinants. They represent much more unspecific interventions such as general education to improve knowledge. In contrast, one extracted determinant (Prescribing errors) could not be matched to a specific intervention. Although, it is obvious that studies to reduce prescribing errors were performed, they may not have been aimed at enhancing adherence to treatment.

Our matching list allows for the assessment of congruency between interventions and patient determinants in published trials. Under the prerequisite that a causal relationship exists between our interventions and corresponding patient determinants, our list may help to assess the quality of published studies and their results (Nieuwlaat et al., 2014). Furthermore, our matching list may be useful to develop interventions and to plan trials to assess the effectiveness of interventions with respect to modifiable and unmodifiable patient determinants.

We acknowledge some limitations. First, we applied a very specific search strategy to identify the most relevant literature. A systematic approach with broader search terms and additional databases might have yielded more articles, however, may not have yielded additional items of interventions or patient determinants. The 85% overlapping between our determinants and those retrieved from a systematic review of reviews (Kardas et al., 2013) reinforces this assumption. Second, we did not consider the effectiveness of the interventions, the frequency of the patient determinants, nor the impact size of the patient determinants on adherence. Consequently, matching interventions to patient determinants based on our results does not guarantee for a successful adherence intervention. Other concepts may be important to consider: determinants may be different for each phase of medication adherence: initiation, implementation, and persistence (Vrijens et al., 2012; Kardas et al., 2013). The current literature lacks the information about which determinant is associated to each of the three phases. Third, our final matching list was not externally validated. However and in line with others, the existence of ~40 different determinants seems plausible.

Our study has some strengths. First, we based our selection on published models and theories, and previously proposed categories. Consequently, our matching list represents a robust framework in line with underlying theories. Second, reliability was given from two independent investigators for extraction and categorization reaching substantial agreement. Third, the exclusion of reviews with focus on specific diseases, populations or other criteria guarantees a broad applicability of the matching list.

## Conclusion

Matched interventions and patient determinants in common categories are needed to assess the congruency between interventions and patient determinants in published trials on medication adherence. Our matching list may be useful to develop interventions in trials that investigate the effectiveness of adherence interventions. Application of this list will show its practicability and may initiate its refinement and further development into a practical tool. To be successful, interventions in medication adherence should target current modifiable patient determinants and be tailored to the unmodifiable patient determinants. Modifiable and unmodifiable determinants need to be assessed at inclusion of intervention studies to identify the patients most in need of an adherence intervention.

## Author contributions

All authors contributed to the design of the study. SA and IA performed the analysis and drafted the manuscript. RN, BV, and KH critically revised the work prior to submission. All authors approved the manuscript in its final version for submission and agreed to be accountable for the work presented.

## Funding

This study was funded by the University of Basel, Switzerland.

### Conflict of interest statement

The authors declare that the research was conducted in the absence of any commercial or financial relationships that could be construed as a potential conflict of interest.
